# New Ibuprofen Cystamine Salts With Improved Solubility and Anti‐Inflammatory Effect

**DOI:** 10.1002/open.202400206

**Published:** 2024-10-14

**Authors:** Simay Denizkusu, Ece Sabuncu, Hande Sipahi, Duygu Avci

**Affiliations:** ^1^ Department of Chemistry Bogazici University 34342 Bebek Istanbul Turkey; ^2^ Department of Pharmaceutical Toxicology Faculty of Pharmacy Yeditepe University 34775 Istanbul Turkey

**Keywords:** cystamine, drug design, ibuprofen, inflammation, redox responsive

## Abstract

Two novel ibuprofen cystamine salts (IBU‐CYS 1 and IBU‐CYS 2) are synthesized by coupling the anion of ibuprofen with cystamine dihydrochloride in 1 : 1 and 2 : 1 ratio to improve the solubility and bioavailability of ibuprofen. The salts are characterized by ^1^H NMR, FT‐IR and UV‐Vis spectroscopy, differential scanning calorimetry (DSC), thermogravimetry (TGA, DTA) and X‐ray diffraction measurements. IBU‐CYS 1 and IBU‐CYS 2 show higher solubility (6.11 and 7.81 mg/mL) compared to ibuprofen (0.04 mg/mL) in water. IBU‐CYS2 was encapsulated into 2‐hydroxyethyl methacrylate: poly (ethylene glycol) acrylate hydrogels for enhanced delivery. The *in vitro* studies in PBS (pH 7.4) indicate that the salts are effective in relieving inflammatory responses induced by lipopolysaccharide in RAW264.7 macrophage cells (nitrite inhibition percentages of IBU‐CYS 1, IBU‐CYS 2 and ibuprofen: approximately 34.29, 27.03 and 31.50 respectively) while indicating no cytotoxicity. Therefore, these salts may be promising candidates for the development of effective formulations of this drug.

## Graphical abstract

We describe the design, synthesis and evaluation of two novel ibuprofen cystamine salts (IBU‐CYS 1 and IBU‐CYS 2) to improve the solubility, bioavailability and anti‐inflammatory activity of ibuprofen.

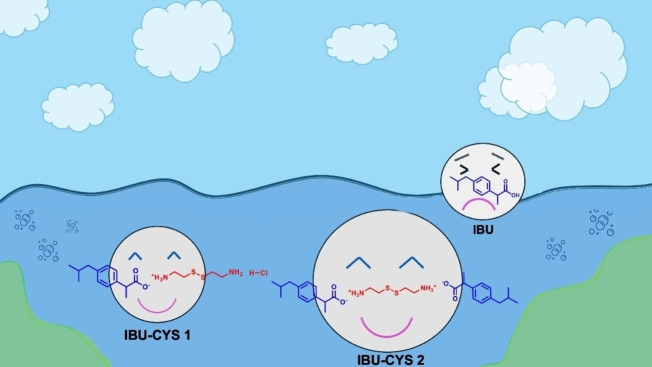

## Introduction

Ibuprofen (IBU) is a non‐steroidal anti‐inflammatory drug (NSAID) used for the treatment of fever, pain and inflammation. It shows therapeutic effects by inhibiting cyclooxygenase‐1 (COX‐1) and cyclooxygenase‐2 (COX‐2), resulting in the inhibition of prostaglandins which are involved in the development of pain, inflammation, and fever.[^1^, ^2^] However, its relatively low solubility (0.076 mg cm^−3^ in water), particularly in the acidic environment of the stomach (0.024 mg cm^−3^ at pH 2.2) constitute a handicap, slowing its pharmacological action.[^3^, ^4^] Therefore, research efforts are underway to develop new derivatives with similar pharmaceutical activity but increased solubility and bioavailability and thus – substances with a rapid pain relief.[^3^, ^4^, ^5^, ^6^, ^7^, ^8^, ^9^, ^10^, ^11^, ^12^] This article is intended as a contribution to these efforts.

Bioavailability is critical for the therapeutic efficacy of a drug. It determines the proportion of the active substance available in the body in relation to its amount in the drug.^[5]^ Hence, the lower the bioavailability, the higher will be the required dose; however excessive drug concentrations can produce toxicity and side effects, especially if the drug is not targetable to the treatment organ/tissue.^[5]^ For example, in the case of ibuprofen, which is conventionally administered orally over a period of time, serious gastrointestinal side effects are observed. Factors affecting bioavailability include mode of administration, drug's physicochemical properties and solubility which effects absorption. Several studies have also shown that faster‐absorbed formulations lead to faster onset of analgesia.^[13]^

Salt formation of active pharmaceuticals is a very well‐known method to optimize their physicochemical and biological properties,[^13^, ^14^] effecting bioavailability. Properties such as solubility, dissolution rate, hygroscopicity and stability can be influenced by salt formation.^[13]^ Although salts with different counterions may improve the shortcomings of active pharmaceuticals, a correlation between salt structure (counterions) and properties of active pharmaceuticals was not accurately investigated. The selection of counterions depends on their toxicological suitability, which is proved by their earlier usage in FDA approved drugs.[^15^, ^16^]

Salts of ibuprofen with ethylamine, diethylamine, triethylamine and ethylene diamine as counter‐ions were synthesized to determine the effect of these counter‐ions on the permeation of ibuprofen across a lipophilic membrane.^[6]^ The highest flux was measured for ibuprofen trimethylamine.^[6]^ Other ibuprofen salts, such as ibuprofen lysine and ibuprofen arginate, were prepared and they were found to be more rapidly absorbed than free ibuprofen acid.[^10^, ^11^] Another ibuprofen salt, sodium ibuprofen dihydrate (ibuprofen sodium), manufactured by Hoffmann‐La Roche Ltd, was recently shown to be bioequivalent to the lysine and arginate salt forms.^[17]^ Poloxamers have been used to enhance dissolution and bioavailability of poorly water‐soluble drugs, including ibuprofen.[^17^, ^18^] Ibuprofen isobutanolammonium (marketed as Ginenorm) is a salt developed for the treatment of inflammation associated with vaginal infections. Studies have also proven that ibuprofen isobutanolammonium has synergistic antimicrobial action when used in conjunction with other antimycotic and antibacterial drugs.^[7]^ Ibuprofen phosphatidylcholine salts were prepared to obtain improved solubility, which may cause improved bioavailability.^[19]^ To avoid the gastrointestinal side effects due to oral administration of ibuprofen, a transdermal local drug delivery system was developed, which thereby enhances therapeutic efficacy and furthermore has a more rapid onset.^[20]^ In order to increase skin permeability of ibuprofen it is converted to ionic liquids with different cationic counterions such as tetrabutylphosphonium, lidocaine, tetraalkylammonium and tetraalkylphosphonium.[^21^, ^22^, ^23^]

In this study, we report the synthesis and characterization of two novel cystamine salt derivatives of ibuprofen to increase its solubility and bioavailability. Both cysteamine and its oxidized form cystamine have protective effects in cells and tissues and mitigate oxidative stress and inflammation and upregulate neuroprotective pathways.^[24]^ Cystamine derivatives contain disulfide linkages, therefore may exhibit redox responsivity.[^25^, ^26^, ^27^] They are stable against hydrolysis in the body but are prone to selective cleavage in the reducing environment of tumor tissue and intracellular compartments through thiol–disulfide exchange reactions.^[28]^ Therefore, the synthesized salts may target inflamed tissues better compared to ibuprofen so that side effects of ibuprofen can be alleviated. The salts were characterized using NMR and FTIR spectroscopy, thermogravimetry (TGA, DTA), differential scanning calorimetry (DSC) and X‐ray diffraction (XRD). A solubility study was also conducted to assess any improvements in solubility of the parent drug. Also, one of these drugs was physically incorporated into a 2‐hydroxyethyl methacrylate (HEMA):poly (ethylene glycol) diacrylate (PEGDA, M_n_=575 D) hydrogel matrix to create a control release base to increase bioavailability and decrease gastric side effects.

## Results and Discussion

### Synthesis and Characterization of Ibuprofen Salts

**Scheme 1:**
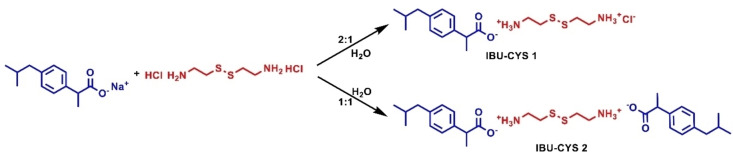
Synthesis of ibuprofen salts.

**Figure 1:**
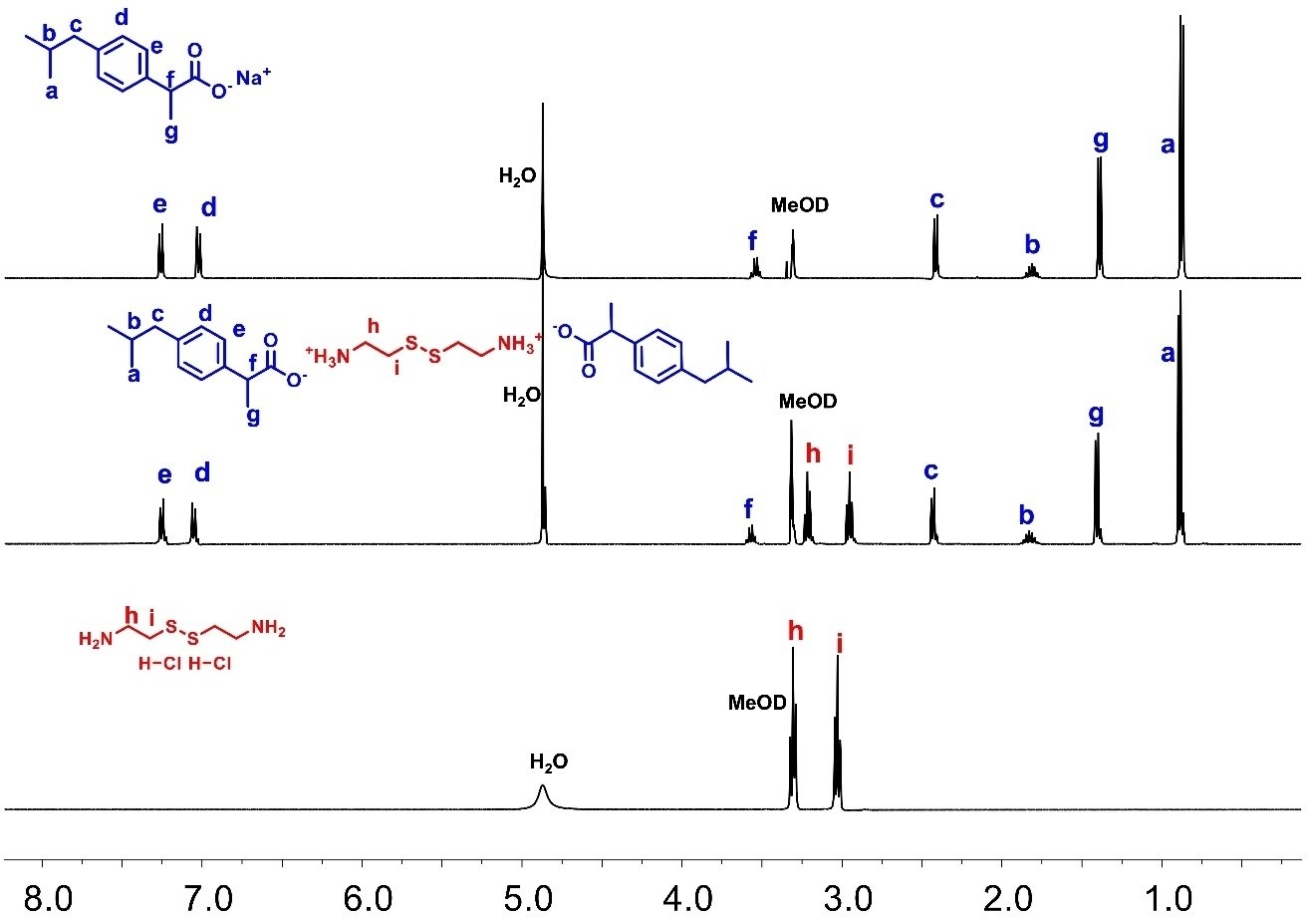
^1^H NMR spectra of ibuprofen sodium salt, cystamine dihydrochloride and IBU‐CYS 2 in MeOD.

**Figure 2:**
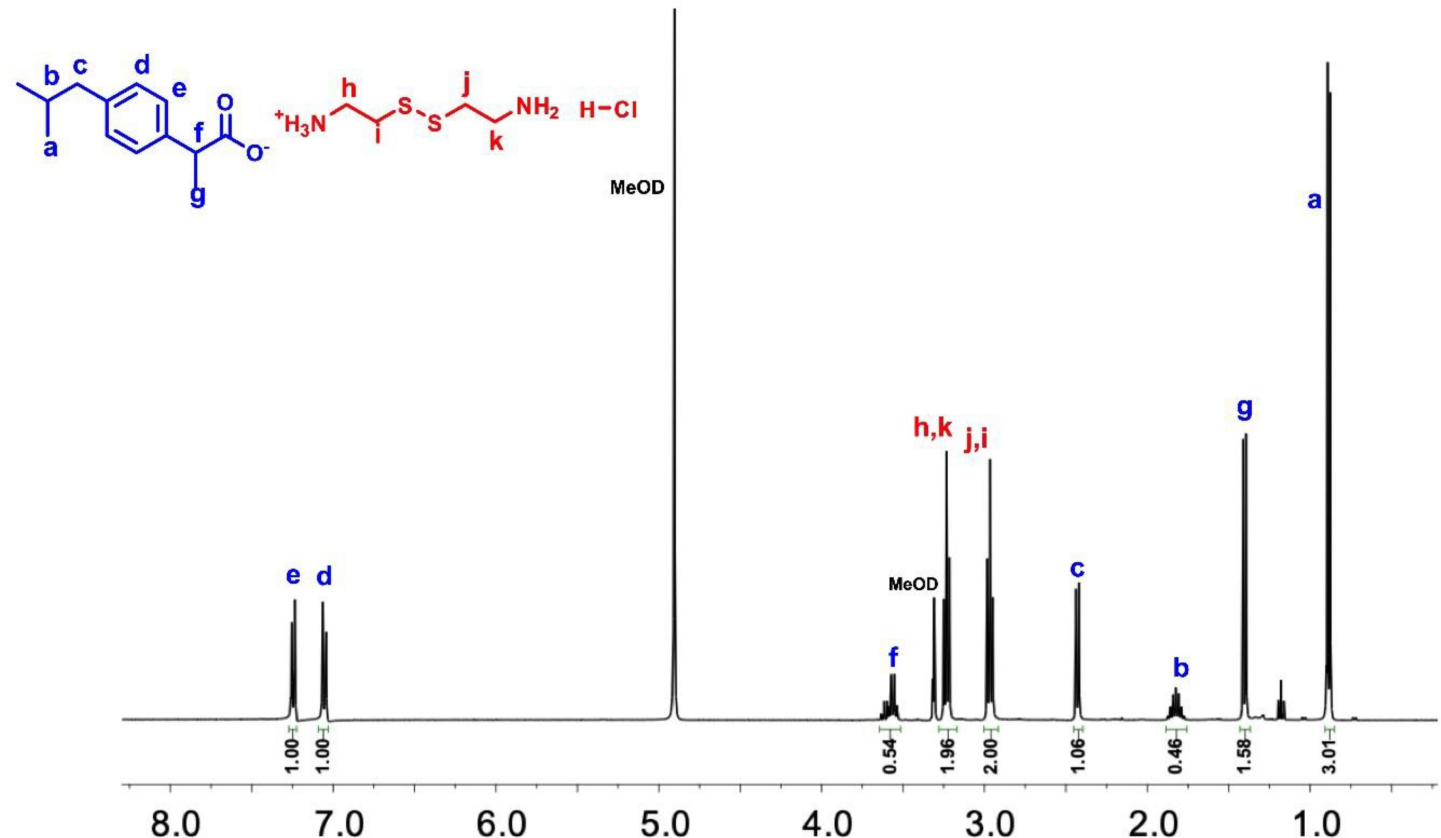
^1^H NMR spectrum of IBU‐CYS‐1 in MeOD.

Two novel ibuprofen salts (IBU‐CYS 1 and IBU‐CYS 2) were successfully synthesized by an electrostatic interaction mechanism between ibuprofen sodium salt and cystamine dihydrochloride in 1 : 1 and 2 : 1 mole ratios as white crystalline solid with a reasonable yield (~50–70 %) (Scheme 1). Their structures were investigated by NMR and IR spectroscopy (Figure 1–Figure 4, Figure S1–Figure S3). The ^1^H NMR spectrum of IBU‐CYS 1 and IBU‐CYS 2 revealed aromatic protons at 7.00 and 7.18 ppm and two methyl protons at 0.83 and 1.34 ppm as doublets of doublets and a methine proton as a multiplet due to proton/proton vicinal coupling (Figure 1, Figure 2, Figure S1, Figure S2). The formation of cystamine salts was supported by the shift of cystamine protons, two triplets at ca. 2.87 and 3.25 ppm, toward higher field. Also the quartet from methine proton next to carboxylate is shifted slightly toward lower field (3.50 ppm). When the ^1^H NMR spectra of the salts were recorded in CDCl_3_, the protonated amino groups were observed at 3.50 ppm as a broader peak compared to the others (Figure S2).

**Figure 3:**
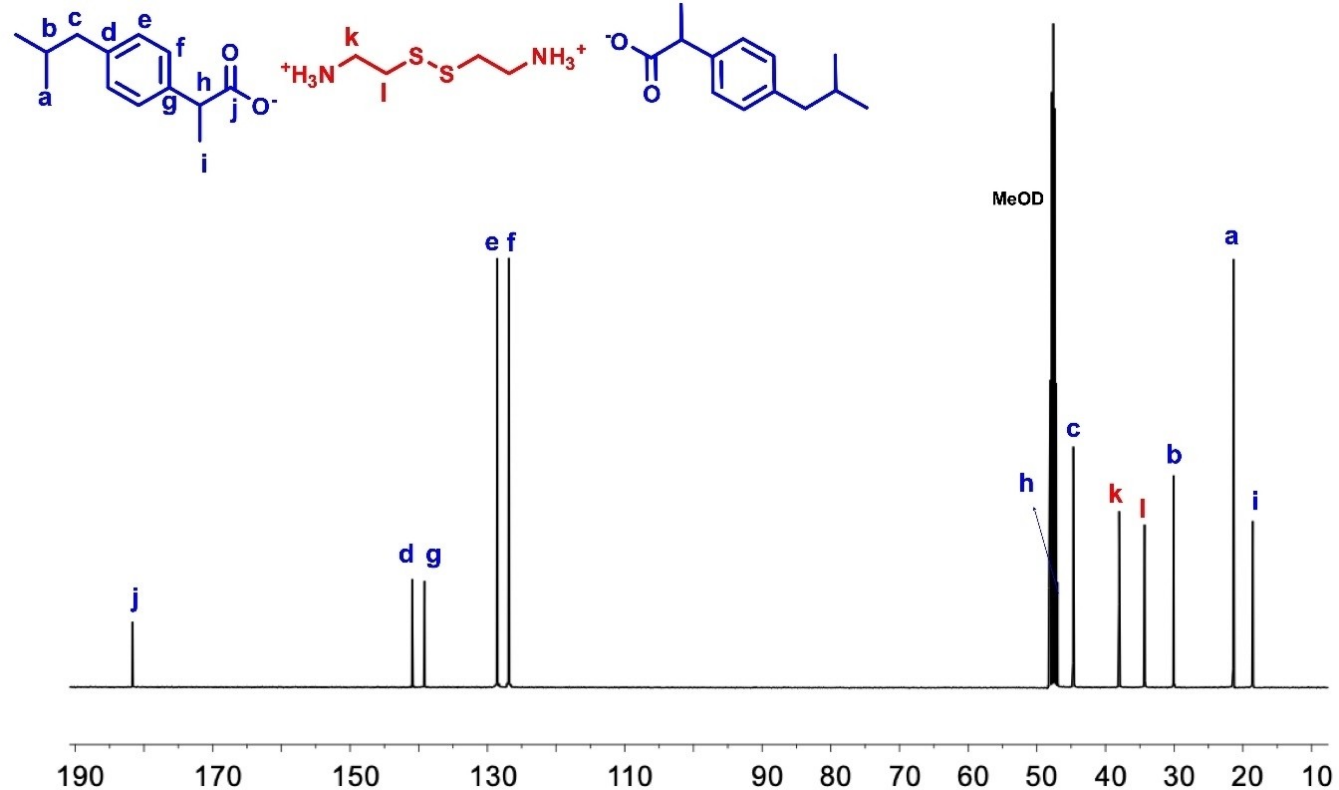
^13^C NMR spectrum of IBU‐CYS 2 in MeOD.

**Figure 4:**
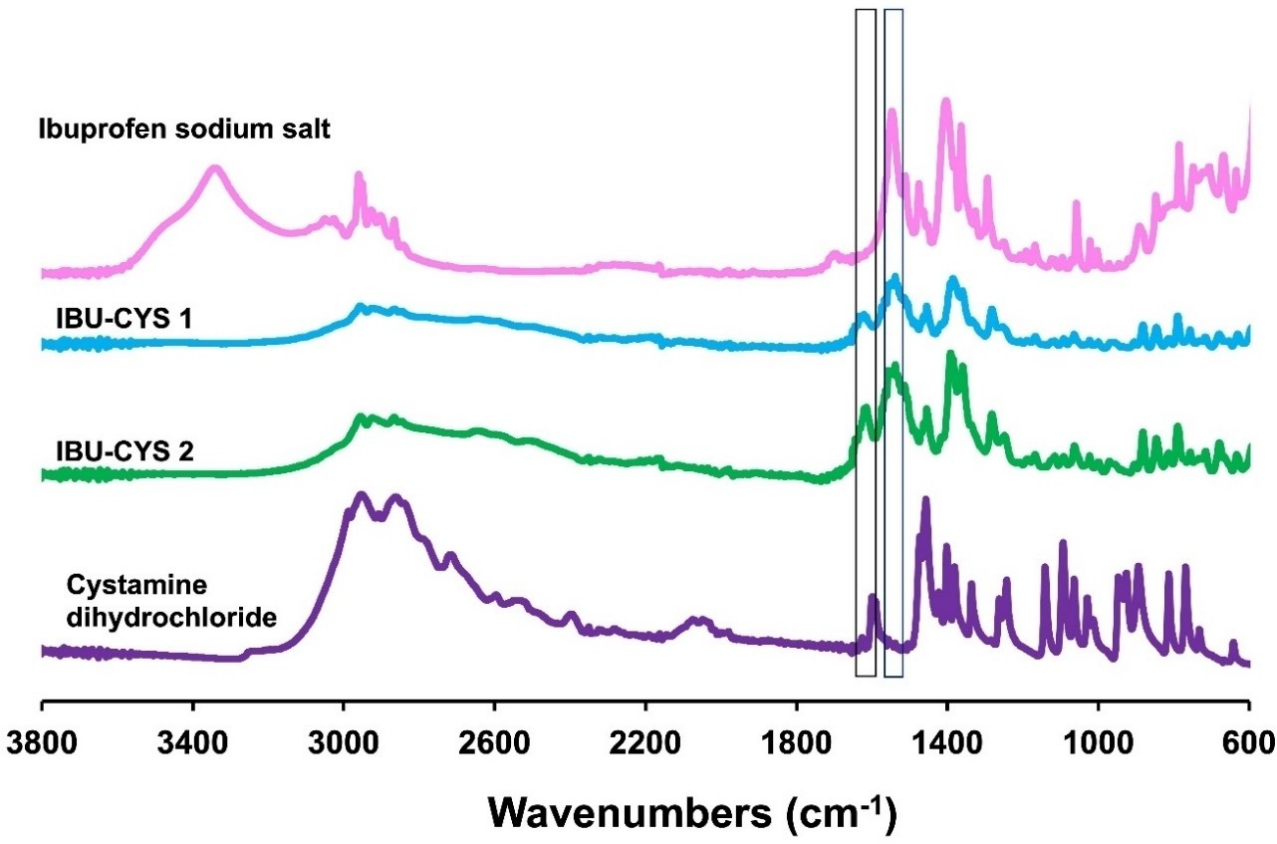
FTIR spectra of ibuprofen sodium salt, cystamine dihydrochloride, IBU‐CYS 1 and IBU‐CYS 2.

^1^H NMR spectra of the salts were also used to determine the exact ratio of cystamine to ibuprofen. In the ^1^H NMR spectrum of IBU‐CYS 1 and IBU‐CYS 2, the aromatic protons (e or d) were integrated with respect to CH_2_ protons (h, i, j or k) of cystamine. For example, in IBU‐CYS 2 the ratio of e to h protons is 1 : 1, indicating formation of difunctionalized product. However, in IBU‐CYS 1 this ratio is 1 : 2 (Figure 2, Figure S2). In the ^13^C NMR spectrum of IBU‐CYS 2, peaks belonging to cystamine carbons were observed at 34.30 and 38.02 ppm, while aromatic carbons of ibuprofen were observed between 126 and 140 ppm (Figure 3). The FTIR spectra of the synthesized salts were compared to that of the precursors and confirmed the ionic structure of the synthesized compounds (Figure 4). The salts’ spectra showed an asymmetric NH_3_^+^ bending at 1615 cm^−1^ and a symmetrical bend at 1514 cm^−1^.^[29]^ The carboxylate anion (COO^−^) absorbs strongly at around 1563 and 1359 cm^−1^ due to asymmetric and symmetric stretching vibrations.

**Figure 5:**
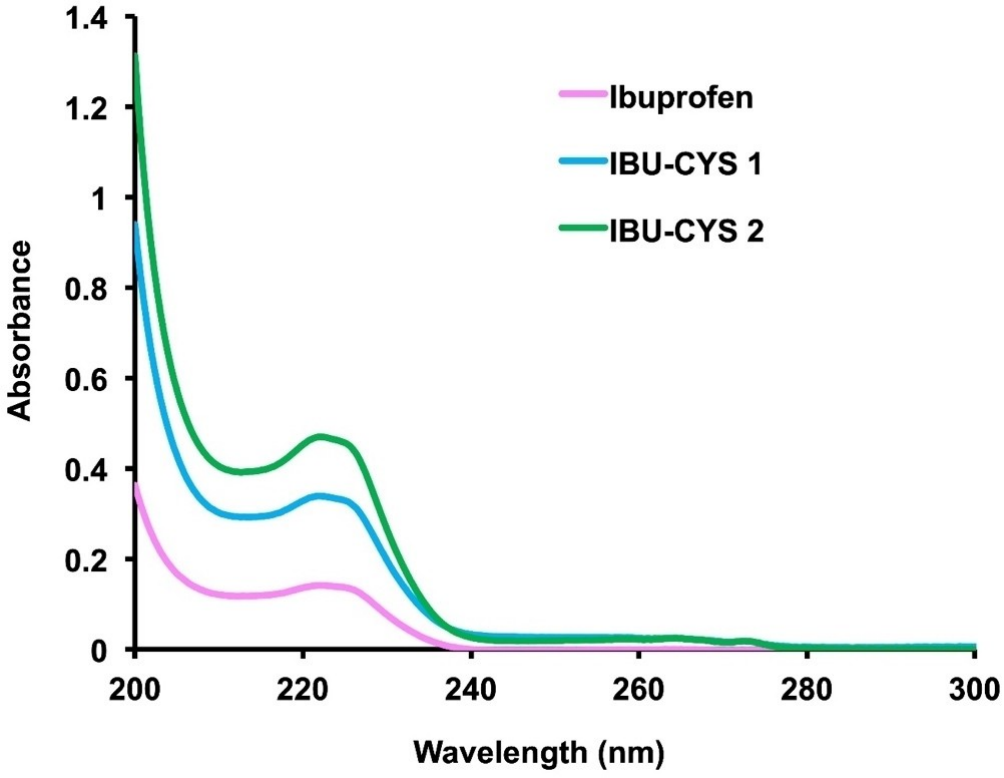
UV‐Vis spectra of IBU, IBU‐CYS 1 and IBU‐CYS 2 in water (3.4×10^−^5 M).

Table 1 summarizes the solubilities of IBU‐CYS 1 and IBU‐CYS 2 and IBU as reference, in various solvents. The synthesized salts exhibit enhanced solubility in polar solvents compared to non‐polar solvents. They show similar solubility behavior to IBU in highly polar (methanol), slightly polar (CHCl_3_ and THF) solvents; however, these salts diverge from ibuprofen in terms of solubility in non‐polar solvents like hexane. The solubility of IBU‐CYS 1 and IBU‐CYS 2 in water was found to be 6.11 and 7.81 mg/mL respectively (Table 2). This is a significant improvement over the solubility of IBU which was found to be 0.04 mg/mL under the same conditions. These salts have lower solubility than ibuprofen sodium salt (>100 mg/mL) and ibuprofen isobutanolammonium salt (315.201 mg/mL) in water.[^7^, ^20^] The solubility of the synthesized salts increased in PBS (pH=7.4) to 7.48 and 9.24 mg/mL, which are 3.81 and 4.71 times higher than IBU. These values were comparable to those of L‐valine ester ibuprofenates in pH 7.4 buffer (1.45 to 7.07 times higher than that of ibuprofen).^[30]^ Thus one can conclude that these values are also higher than tetrahexylammonium, trihexyltetradecylphosphonium and didecyldimethylammonium ibuprofenates.^[30]^ The UV‐vis spectra of the salts show the maximum absorbance at 223–224 nm, similar to IBU, indicating the π→π* electronic transition (Figure 5). Their absorbance vs. concentration plots in water were shown in Figure S4.

**Table 1:** Solubilities of IBU, IBU‐CYS 1 and IBU‐CYS 2.

Salt/IBU	H_2_O	MeOH	EtOH	(C_2_H_5_)_2_O	CHCl_3_	DCM	THF	Hexane
IBU	–	+	+	+	+	+	+	+
IBU‐CYS 1	+	+	+	±	+	–	+	–
IBU‐CYS 2	+	+	+	±	+	–	+	–

**Table 2:** Solubility Table of IBU‐CYS salts and ibuprofen in both water and PBS expressed in mg/mL.

Salt/IBU	Water	PBS
IBU	0.04	1.96
IBU‐CYS 1	6.11	7.48
IBU‐CYS 2	7.81	9.24

**Figure 6:**
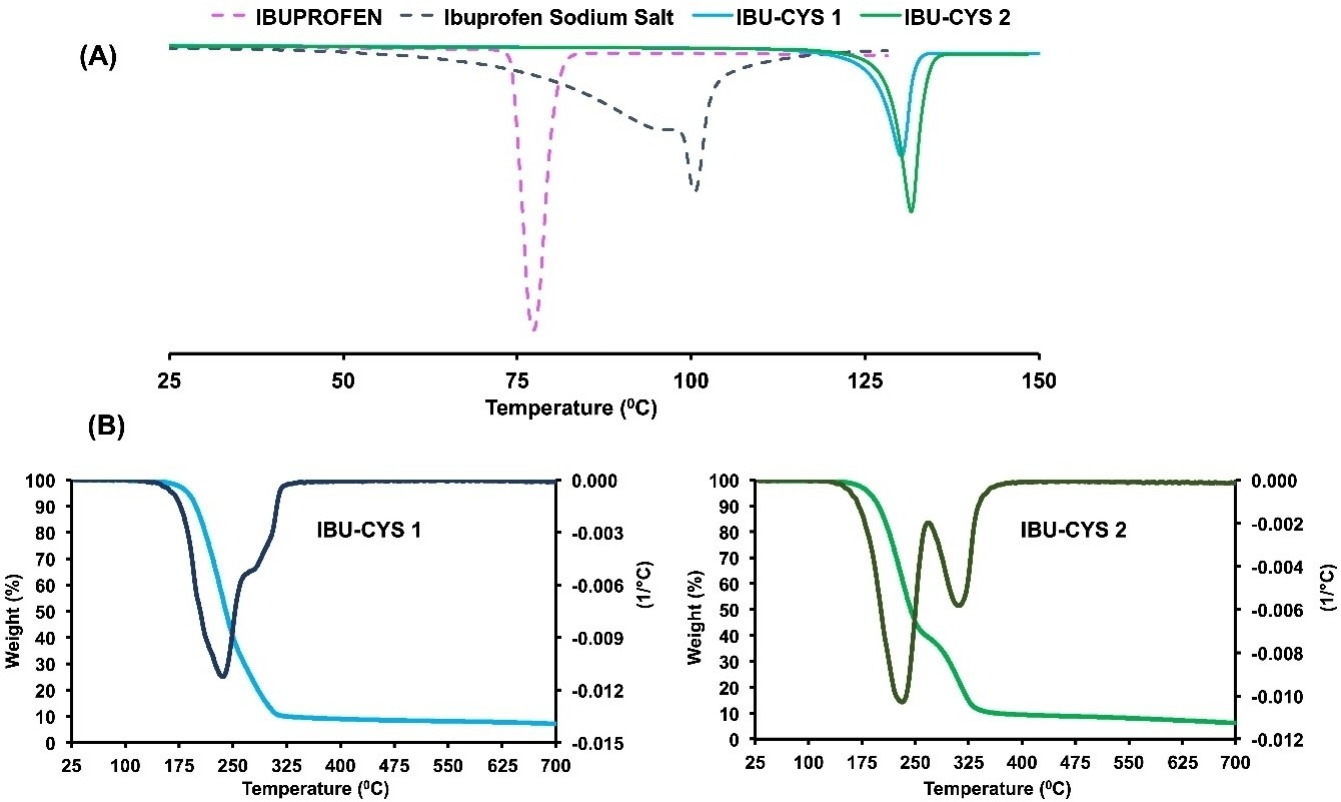
**(**A) DSC spectra of IBU, IBU sodium salt, IBU‐CYS 1 and CYS 2, (B) TGA and DTA spectra of IBU‐CYS 1 and IBU‐CYS 2.

The thermal properties of the salts were investigated using DSC and TGA/DTA. The DSC plots of IBU and the synthesized salts are given in Figure 6. IBU shows an endotherm at 77.36 °C, corresponding to its melting point (75–77.5 °C).^[31]^ On the other hand, DSC plots of IBU‐CYS 1 and IBU‐CYS 2 showed very sharp endotherms at 131.11 and 131.62 °C respectively, indicating their crystalline structures. The melting points were found to be independent of their structures; both are 53–54 °C higher than IBU. The ibuprofen sodium salt exhibited an endotherm at temperature about ~110 °C, attributed to dehydration (Figure 6A) and another peak at 170 °C due to melting.^[31]^

Thermal stabilities of the salts were investigated by TGA/DTA under nitrogen atmosphere (Figure 6). The thermal degradation of IBU‐CYS 1 occurred in two steps starting from 177 °C, a DTA peak appears with maximum at 236 °C. IBU‐CYS 2 also showed two distinct steps of thermal degradation at temperatures of approximately 172 and 220 °C. These steps showed mass loss percent of 60 and 89 %, accompanied by endothermic DTA peaks at 233 and 310 °C respectively. The decomposition of ibuprofen takes place at approximately 150 °C in one step under similar conditions.[^32^, ^33^] Cystamine dihydrochloride decomposes between 273.8 and 328.0 °C giving a char yield of 23.2 %.^[34]^ These results indicated that the synthesized salts are more stable than ibuprofen and less stable than cystamine dihydrochloride.

XRD patterns of the IBU‐CYS 1 and IBU‐CYS 2 were compared with those of their starting materials, ibuprofen sodium salt and cystamine dihydrochloride (Figure S5 and S6). Cystamin dihydrochloride exhibits a main peak at 2θ 27°, while pure ibuprofen sodium salt has peaks at 2θ 17°.^[35]^ The disappearance of these main peaks in the IBU‐CYS 1 and IBU‐CYS 2 diffractograms revealed the formation of new salts, with complicated and sharp diffraction peaks between 2θ 20–40° indicating their crystalline structures.

### In Vitro Release Studies

Encapsulation of drugs in a hydrogel matrix enables reduction of drug dosage and minimize potential side effects.[^36^, ^37^] Therefore, hydrogels of biocompatible monomers HEMA and PEGDA were prepared using photopolymerization to accommodate IBU‐CYS 2. The surface morphology of the hydrogels was investigated by SEM/EDX and compared with the reference PEGDA : HEMA hydrogels (Figure S7). The PEGDA : HEMA : IBU‐CYS 2 hydrogels were observed to have less porous structure compared to those of the reference due to deposited IBU‐CYS 2. The EDX and mapping results showed the presence of sulfur in PEGDA : HEMA : IBU‐CYS 2 hydrogels and correlates well with the amount of IBU‐CYS 2 loaded.

**Figure 7:**
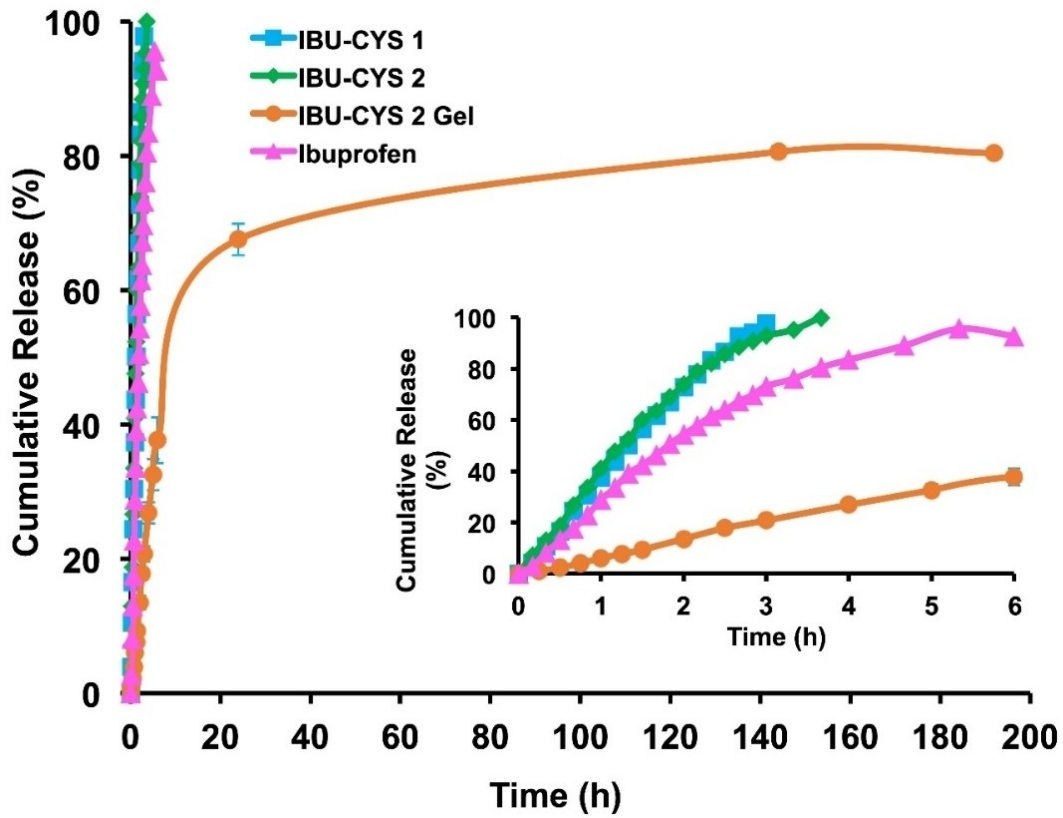
*In vitro* release results in PBS.

The release of IBU‐CYS 2 encapsulated in HEMA : PEGDA hydrogel was compared with diffusion of the synthesized salts out of a reasonably concentrated solution through a dialysis membrane, all in PBS solution at physiological pH 7.4. Figure 7 shows the results. It was clearly observed that the diffusion of the salts occurred more quickly than ibuprofen, probably because the salts were better dissolved in the dialysis bag. The release of IBU‐CYS 2 from hydrogel encapsulation, also behind the dialysis membrane, occurs slowly. The drug release was rapid within the first 6 h (40 %), lasted for about 1 week and the cumulative release was ~80 %.

### In Vitro Cytotoxicity of the Ibuprofen Salts

Prior to the evaluation of anti‐inflammatory activity, non‐toxic concentrations of samples were determined by cell viability of 70 % and above using MTT assay, and both salt forms were observed to be non‐cytotoxic (Table 3) in the concentration range we studied (12.5–100 μm).

**Table 3:** Effects of different salt forms of ibuprofen on the viability of RAW264.7 macrophage cells and the effects of the different salt forms of ibuprofen on nitrite levels and % nitrite inhibition in RAW 264.7 cells stimulated with 1 μg/mL LPS.

Groups	Dose (μM)	Cell Viability (%)	Nitrite Level (μM)	Nitrite Inhibition (%)
Ctrl		112.44±3.07	2.42±0.20	–
LPS		100.00±0.05	60.92±0.79	–
L‐Name	100	92.85±2.05	30.93±0.87**	49.23±1.71
Ibuprofen	100	93.87±0.35	41.73±1.59**	31.50±2.53
Indomethacin	100	94.12±1.39	29.88±0.72**	50.95±0.62
IBU‐CYS 1	12.5	105.86±4.42	51.14±1.33**	16.03±2.91
	25	100.99±3.50	47.34±0.49**	22.28±0.86
	50	93.74±2.83	44.39±1.33**	27.15±1.53
	100	89.29±3.46	40.03±0.64**	34.29±1.18
IBU‐CYS 2	12.5	102.27±0.60	54.41±0.53*	10.66±1.89
	25	97.91±3.34	50.44±0.99**	17.20±1.56
	50	92.61±2.46	48.13±1.71**	20.99±2.43
	100	87.63±1.15	44.45±0.26**	27.03±0.84

Ctrl: Control group treated with DMEM; LPS: Control group only stimulated with LPS; LPS: Lipopolysaccharides from *E. coli*; IND: Indomethacin (100 μM). Statistically significant differences were indicated for each compound vs. LPS (*p<0.05, **p<0.001.)

### In Vitro Evaluation of the Anti‐Inflammatory Effect

**Figure 8:**
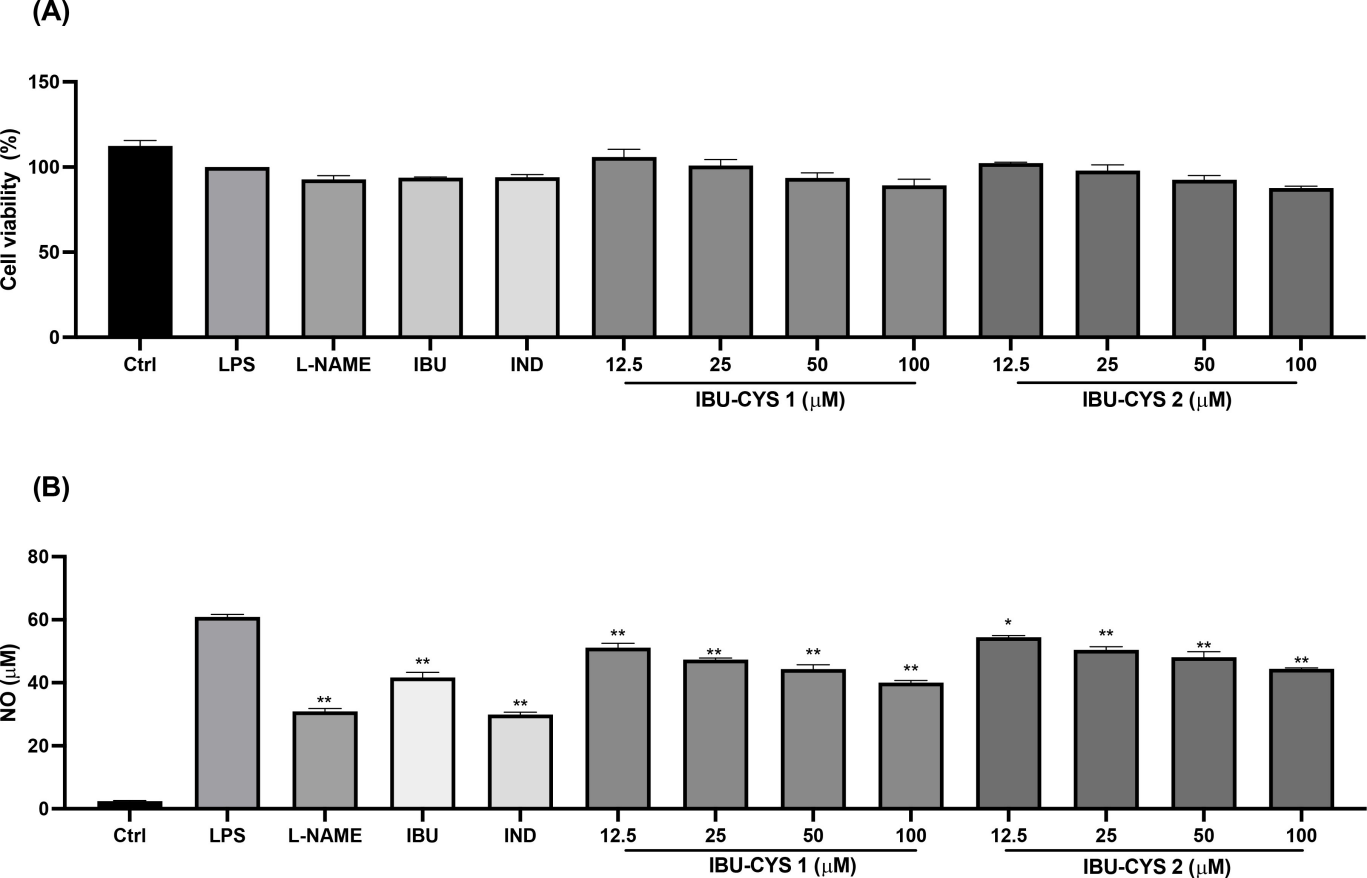
The effects of IBU‐CYS 1 and IBU‐CYS 2 at different doses (12.5, 25, 50 and 100 μM) on cell viability (A), on nitrite production (B) on LPS stimulated RAW 264.7 cell. Ctrl: Control group treated with DMEM; LPS: Control group only stimulated with LPS; LPS: Lipopolysaccharides from *E. coli*; IND: Indomethacin (100 μM). Statistically significant differences were indicated for each compound vs. LPS (*p<0.05, **p<0.001).

The ability of the ibuprofen salts to inhibit LPS‐induced nitrite production in RAW 264.7 cells was evaluated and compared with those of L‐name (N(G)‐Nitro‐L‐arginine methyl ester) (100 μM) which is a direct inhibitory agent of nitrite oxide and indomethacin (100 μM) and ibuprofen (100 μM), serving as reference compounds. Treatment with LPS (1 μg/mL) led to a significant increase in nitrite levels in the cell culture supernatant (Table 3, Figure 8). NO production after LPS stimulation increased to 60.92±0.79 μM in the untreated cells. However, both salt forms exhibited a concentration‐dependent reduction in LPS‐induced nitrite production, as shown in Table 3. The treatment of L‐name (100 μM) and indomethacin (100 μM) significantly inhibited nitrite oxide production as 49 % and 50 % respectively (p<0.001). In comparison of anti‐inflammatory activity of L‐name and indomethacin, ibuprofen showed less anti‐inflammatory effect. This is due to the information from a study that revealed the use of lower doses than 200 μM of ibuprofen produces effect out therapeutic range therefore have slight anti‐inflammatory effect.^[38]^ Moreover, nitrite inhibition of IBU‐CYS 1 and IBU‐CYS 2 at their highest studied concentrations was approximately 35 % and 27 % respectively. As a result, ibuprofen cysteine 1 : 1 salt form at 100 μM concentration has shown greater anti‐inflammatory activity in comparison to ibuprofen.

## Conclusions

In this work, we first successfully and quantitatively synthesized two ibuprofen salts with cystamine cations. The synthesized salts showed improved solubility in water and PBS in comparison with both the free acid and sodium salt of ibuprofen. The salts did not affect the cell viability of RAW264.7 macrophages at the concentrations of 12.5–100 μM, similar to ibuprofen. The derivatives showed higher anti‐inflammatory activity compared to ibuprofen. The encapsulation of the salts into a HEMA : PEGDA hydrogel decreased their release rates, which may decrease excess drug concentrations producing toxicity and side effects. Therefore, the salts and their hydrogels may increase therapy efficacy.

## Experimental Section

### Materials

Sodium salt of ibuprofen (α‐methyl‐4‐(isobutyl) phenylacetic acid, >98 %), cystamine dihydrochloride (2,2′‐diaminodiethyl disulfide dihydrochloride, >98 %), 2‐hydroxyethyl methacrylate (HEMA, >99 %), poly (ethylene glycol) diacrylate (PEGDA, M_n_=575 D), 2‐hydroxy‐4′‐(2‐hydroxyethoxy)‐2 methylpropiophenone (Irgacure 2959, 98 %), indomethacin (98.5–100.5 %), (3‐(4,5‐dimethylthiazol‐2‐yl)‐2,5‐diphenyltetrazolium bromide, 98 %) (MTT), lipopolysaccharide (LPS) from *Eschrechia coli* 0111: B4 and the other reagents and solvents were obtained from Sigma Aldrich (St. Louis, Missouri, USA) and used as received without purification. Ibuprofen was a kind gift from Abdi İbrahim İlaç Sanayi & Ticaret A.Ş. Sodium nitrite was purchased from Fluka Chemika‐BioChemika (Buchs, Switzerland). RAW 264.7 mouse macrophages (ATCC® TIB‐71 TM) were obtained from ATCC (Manassas, VA, USA). Dulbecco's Modified Eagle's Medium (DMEM) supplemented with 10 % fetal bovine serum (FBS) and 1 % penicillin (10.000 units/mL) and streptomycin (10.000 mg/mL) were purchased from Gibco, Thermo Fisher Scientific (Waltham, MA, USA).

### Characterization Methods

NMR spectra were recorded on a Varian Gemini 400 MHz spectrometer with deuterated methanol (MeOD) as solvent. IR spectra were obtained using a Nicolet 6700 FT‐IR spectrophotometer. Differential scanning calorimetric (DSC) measurements were performed on a TA Instruments Q250 with a heating rate of 10 °C/min under nitrogen atmosphere. Thermogravimetric analyses (TGA) were performed by using TA Instruments Q500 under nitrogen atmosphere. The samples were heated from room temperature to 700 °C at a rate of 10 °C/min. XRD analyses was studied by X‐ray diffraction (Rigaku D/max‐2200/PC). Scanning electron microscope /Energy dispersive X‐ray analyser (SEM/EDX)(Thermoscientific Quattro S) was used to assess the morphology and elemental identification of hydrogel samples.

### Synthesis of Ibuprofen Salts

#### IBU‐CYS 1

Cystamine dihydrochloride (32.6 mg, 0.145 mmol) was dissolved in 30 mL water (pH=5.65). Ibuprofen sodium salt (33.1 mg, 0.145 mmol) (pH=7.94) dissolved in 3 mL water was added slowly (in 2 h). The resulting solution (pH=6.94) was freeze‐dried and ethanol was added to dissolve the residue. The small amount of undissolved solid was removed and ethanol was evaporated to obtain the product as a white solid in 72 % yield.

^1^H NMR (400 MHz, MeOD, δ): 0.83 (d, 6H, C*H*_3_−CH), 1.34 (d, 3H, C*H*_3_−CH), 1.76 (dt, 1H, (CH_3_)_2_−C*H*−CH_2_), 2.35 (d, 2H, C*H_2_*−Ar), 2.87 (t, 4H, −CH_2_CH_2_S−), 3.14 (q, 4H, −CH_2_CH_2_N−), 3.49 (q, 1H, CH−C=O), 7.00 (d, 2H, Ar−CH), 7.18 (d, 2H, Ar−CH) ppm.

^13^C NMR (100 MHz, MeOD, δ): 17.85 (CH_3_), 21.67 (CH_3_), 30.06 (CH_3_−*C*H), 34.50 (CH_2_−S), 38.10 (CH_2_−NH_3_^+^), 44.89 (Ar−CH_2_), 48.01 (O=C−*C*H), 126.67 (Ar−CH), 128.68 (Ar−CH), 138.91 (Ar−C), 141.00 (Ar−C), 182.00 (C=O) ppm.

FTIR (ATR): 3100–2400 (C−H, NH_3_^+^), 1621 and 1514 (NH_3_^+^), 1563 and 1360 (COO^−^), 1281 (C−H), 787 (C−H) cm^−1^.

#### IBU‐CYS 2

Cystamine dihydrochloride (0.15 g, 0.67 mmol) was dissolved in 5 mL water (pH=5.71). Ibuprofen sodium salt (0.3 g, 1.33 mmol) dissolved in 15 mL water (pH=7.56) was added slowly (in 1 h), and the reaction mixture was stirred at room temperature for 6 h. The resulting solution (pH=7.12) was centrifuged and the collected precipitate was washed with small amounts of water to remove unreacted starting materials and ions adsorbed on the surface. The precipitate was freeze‐dried to obtain the product as a white solid in 51 % yield.

^1^H NMR (400 MHz, MeOD, δ): 0.82 (d, 12H, C*H*_3_−CH), 1.34 (d, 6H, C*H*_3_−CH), 1.77 (dt, 2H, (CH_3_)_2_−C*H*−CH_2_), 2.36 (d, 4H, C*H_2_*−Ar), 2.89 (t, 4H, −CH_2_CH_2_S−), 3.16 (q, 4H, −CH_2_CH_2_N−), 3.50 (q, 2H, CH−C=O), 7.00 (d, 4H, Ar−CH), 7.18 (d, 4H, Ar−CH) ppm.

^13^C NMR (100 MHz, MeOD, δ): 18.54 (CH_3_), 21.37 (CH_3_), 30.08 (CH_3_−*C*H), 34.30 (CH_2_−S), 38.02 (CH_2_−NH_3_^+^), 44.69 (Ar−CH_2_), 48.11 (O=C−*C*H), 126.88 (Ar−CH), 128.71 (Ar−CH), 139.03 (Ar−C), 140.90 (Ar−C), 181.71 (C=O) ppm.

FTIR (ATR): 3100–2400 (C−H, NH_3_^+^), 1615 and 1514 (NH_3_^+^), 1563 and 1359 (COO^−^), 1281 (C−H), 790 (C−H) cm^−1^.

### Synthesis of Hydrogels

IBU‐CYS 2 (0.02 g, 0.035 mmol), PEGDA (0.02 g, 0.037 mmol) and HEMA (0.16 g, 1.22 mmol) were dissolved in water at 10 : 10 : 80 wt % to form precursor solutions for hydrogels. Irgacure 2959 (2 wt % of total monomer weight) was added as a photoinitiator and the mixture were polymerized by exposure to UV light (365 nm) for 30 minutes in a syringe. Since the synthesized salt was encapsulated within the gel matrix to prevent the possibility of release, no washing procedure was carried out. Water was lyophilized.

### Solubility Study

A solubility study was carried out to determine the degree of enhancement in solubility due to the formation of the salts. First, UV absorption spectra of the salts were generated by scanning their aqueous solutions using a JASCO V‐730 UV‐vis spectrophotometer in the range of 200–300 nm. The λ_max_ of the salts were then determined. The calibration curves of the IBU‐CYS salts and ibuprofen were constructed by measuring the absorbance of their solutions prepared at various concentrations at 222 nm. Finally, the saturated solutions of the salts and ibuprofen were prepared using 5 mL of water and 5 mL of PBS separately in vials. The solutions were stirred at 25 °C for 24 h. After this, the solutions were filtered, the filtrates were diluted to measure their absorbances at 222 nm. The solubilities were calculated according to the calibration curves.

### In Vitro Drug Release Studies

The drug release studies of the parent drug ibuprofen, the synthesized salts and salts’ encapsulated HEMA : PEGDA gel forms were done in phosphate buffered saline (PBS). Briefly, the pre‐weighed dry samples and 3 mL PBS were transferred to dialysis membrane (3500 Da) and were placed into a flask containing 150 mL of PBS maintained at 37 °C and stirred at 150 rpm. At predetermined time intervals 4 mL of external solution was removed and equal volume of fresh solution was added to the system. Each sample was analyzed by UV‐vis spectrophotometer (JASCO V‐730) and the amount of released IBU was calculated using the absorbance at 222 nm according to calibration curve. The linear correlation (R^2^=0.999) between the absorption and concentration of IBU was determined using known concentration samples (0.002, 0.004, 0.008, 0.013, 0.018, 0.022, 0.045, 0.067 and 0.1 mg mL^−1^ of IBU in PBS).

### Cell Viability Assay

To determine the effect of IBU‐CYS 1 and IBU‐CYS 2 on cell viability, MTT assay was used. The RAW 264.7 mouse macrophages were cultivated in complete medium, which consisted of DMEM, supplemented with 10 % FBS and 1 % penicillin (10.000 units/mL) and streptomycin (10.000 mg/mL). They were maintained at a temperature of 37 °C with 5 % CO_2_ in a humidified atmosphere.^[39]^ The cells were subcultured once they reached a confluence of 80–90 %. The RAW264.7 cells were initially seeded in 96‐well culture plates at a density of 5×10^4^ cells/well for 24 hours in complete medium. The cells were then treated with various concentrations of the ibuprofen cystamine salts (ranging from 12.5–100 μM) for 24 hours. Then the MTT reagent (0.5 mg/mL in PBS) was added to the cells and incubated for 2 hours at 37 °C with 5 % CO_2_. After removing the culture supernatants, 100 μL of isopropanol was added to each well to dissolve the formazan blue, and the absorbance was measured at 570 nm using a microplate reader (Thermo Fisher Scientific Inc., Waltham, MA, USA). The cell viability assay was conducted three times, and each assay was performed in triplicate (n=9 in three separate experiments). A cell viability of treated cultures less than 70 % compared to untreated control cultures (medium group) is considered as a cytotoxic.^[40]^ The percentage of cell viability was calculated by using the equation:

Cellviability(%)=(As/Ac)×100

where As and Ac show absorbance of the samples and the control, respectively.

### Anti‐Inflammatory Activity

The inhibition of NO production was assessed by measuring nitrite oxide (NO) levels in the cell culture medium using the Griess reagent (0.1 % N‐(1‐Naphthyl)‐ethylenediamine dihydrochloride in 5 % phosphoric acid and 1 % sulfanilamide).^[41]^ RAW 264.7 cells were plated in a 96‐well plate at a density of 5×10^4^ cells/well and incubated for 24 hours at 37 °C with 5 % CO_2_. After pre‐treating the cells with various concentrations of the Ibuprofen cysteine 2 : 1 and Ibuprofen cysteine 1 : 1 (12.5–25–50–100 μM) for 2 hours and stimulating them with 1 μg/mL of LPS for an additional 22 hours, the cell culture supernatant was collected. The cell culture supernatants were collected after 24 hours for NO analysis. The supernatant was mixed with an equal volume of Griess reagent in a 96‐well plate for 10 minutes at room temperature in the dark. The color development corresponding to NO level was assessed at 540 nm using a microplate reader. The nitrite concentrations were determined using a sodium nitrite standard curve. The researchers used Indomethacin (100 mM) as a positive control.^[42]^

### Statistical Analysis

Each experiment was carried out in triplicate. The GraphPad Prism 9 was used for statistical analyses (GraphPad Software, Inc., San Diego, CA; the 8.4.3 version). Following the Tukey post hoc tests, one‐way ANOVA was used to determine group differences.

## Supporting Information

^1^H NMR spectra for IBU‐CYS 1 and IBU‐CYS 2, ^13^C NMR spectrum of IBU‐CYS 1, absorbance vs. concentration plots of IBU‐CYS 1 and IBU‐CYS‐2 in water, XRD data of both salts and cystamine dihydrochloride, SEM images and EDX analysis of hydrogels can be found in the Supporting Information.

## Conflict of Interests

The authors declare no conflict of interest.

1

## Supporting information

As a service to our authors and readers, this journal provides supporting information supplied by the authors. Such materials are peer reviewed and may be re‐organized for online delivery, but are not copy‐edited or typeset. Technical support issues arising from supporting information (other than missing files) should be addressed to the authors.

Supporting Information

## Data Availability

The data that support the findings of this study are available from the corresponding author upon reasonable request.
