# Supplementary material for: New Ibuprofen Cystamine Salts With Improved Solubility and Anti‐Inflammatory Effect
Source: ChemistryOpen. 2024 Oct 14;13(12):e202400206. doi: 10.1002/open.202400206 (PMC11625915; doi:10.1002/open.202400206)
Supplement: Supplementary file 1 — Supporting Information [file OPEN-13-e202400206-s001.pdf]

ChemistryOpen

Supporting Information

New Ibuprofen Cystamine Salts With Improved Solubility and Anti-Inflammatory Effect

Simay Denizkusu, Ece Sabuncu, Hande Sipahi, and Duygu Avci*

Supporting Information

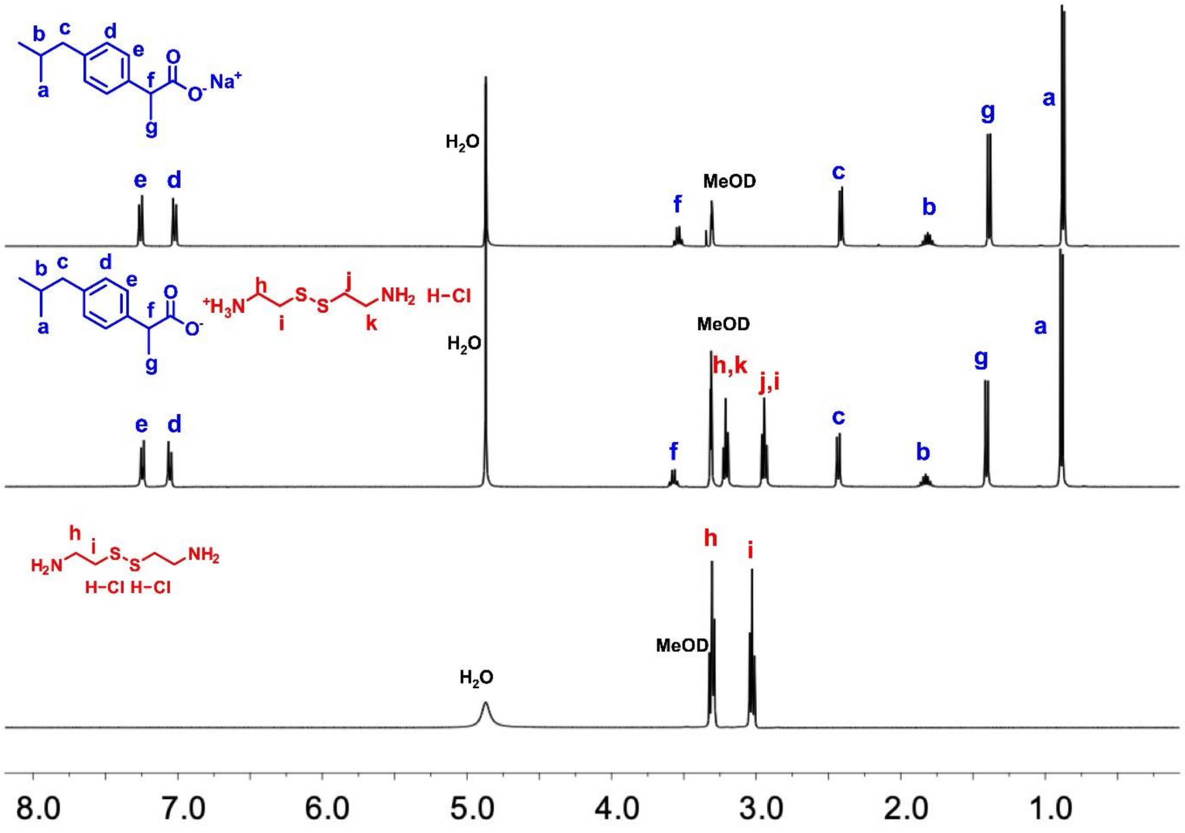

Figure S1. ^{1}H NMR spectra of ibuprofen sodium salt, cystamine dihydrochloride and IBU-CYS 1 in MeOD

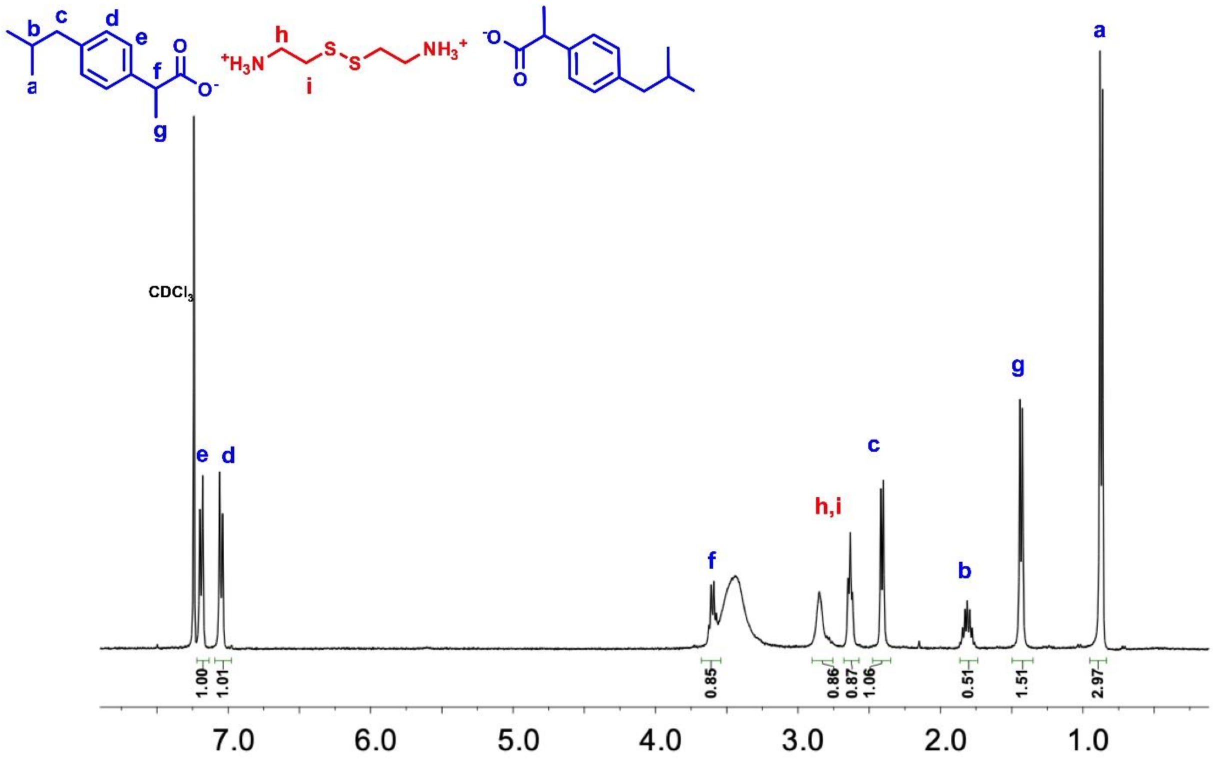

Figure S2. Integrated ^{1}H NMR spectrum of IBU-CYS 2 in $CDCl_3$

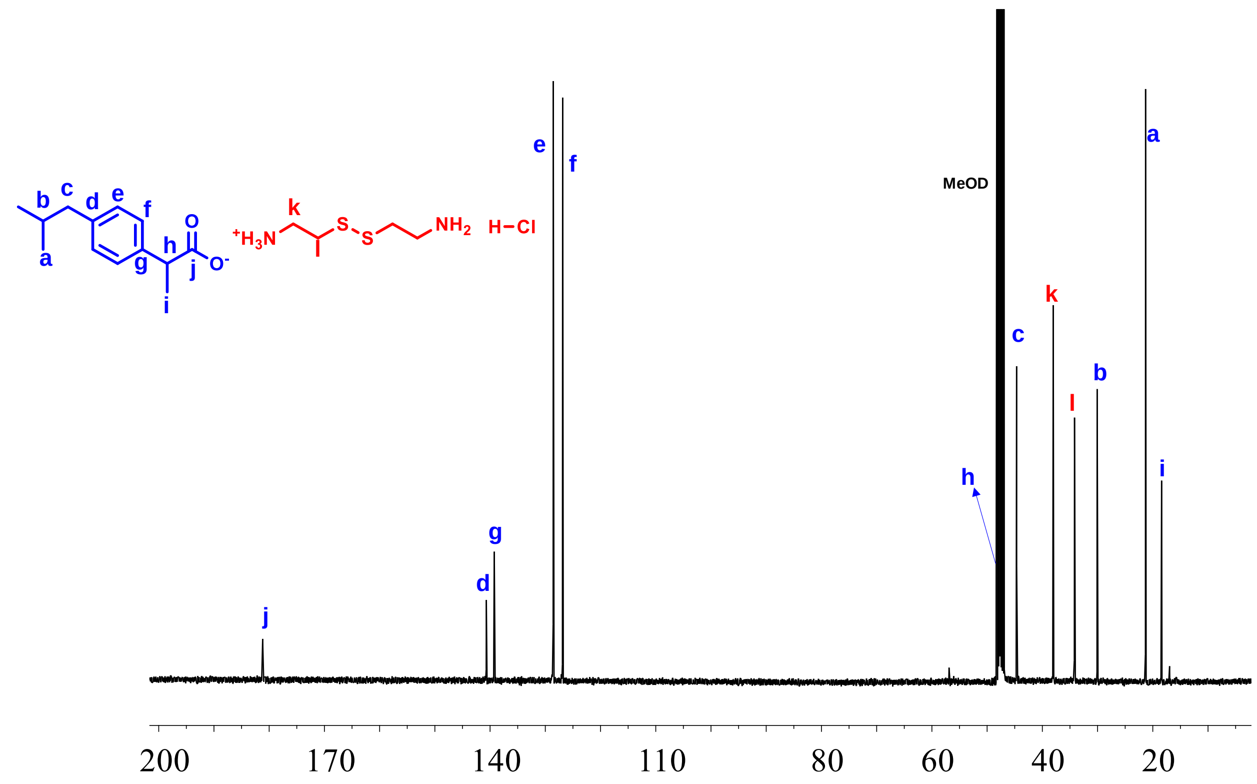

Figure S3. ^{13}C NMR spectrum of IBU-CYS 1 in MeOD

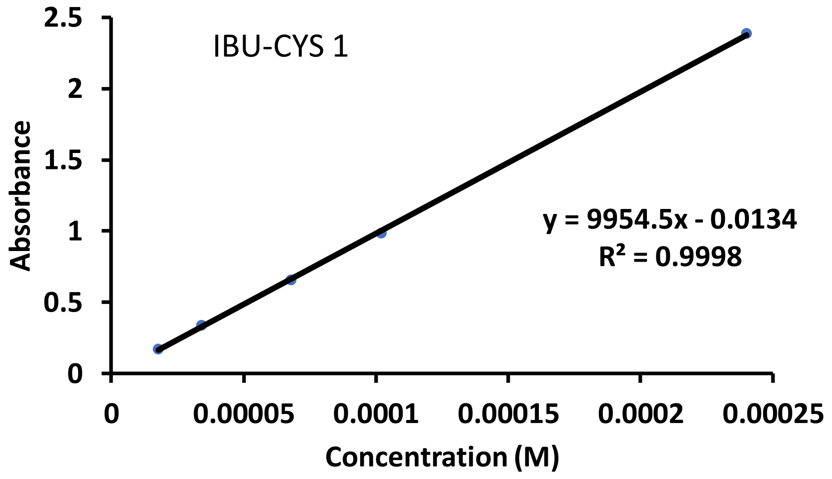

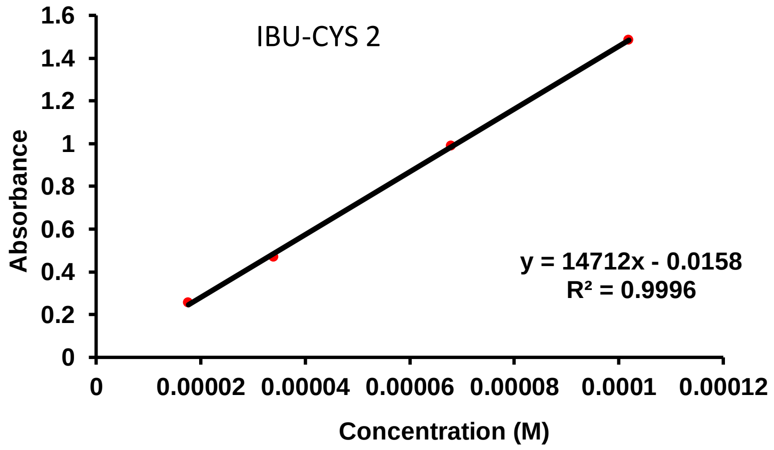

Figure S4. Absorbance vs. concentration plots for IBU-CYS-1 and IBU-CYS 2 in water

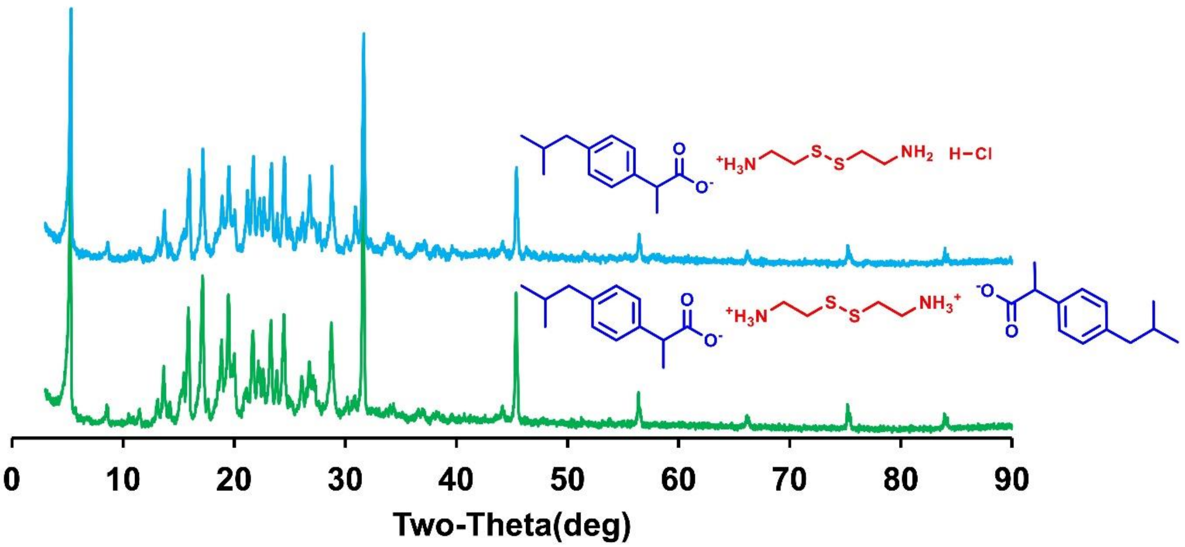

Figure S5. XRD patterns of IBU-CYS 1 and IBU-CYS 2

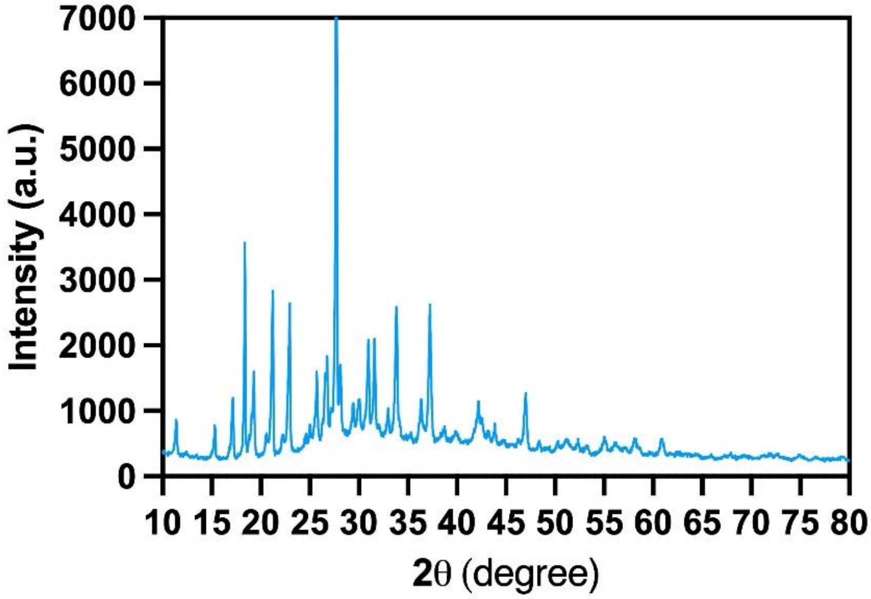

Figure S6. XRD pattern of cystamine dihydrochloride

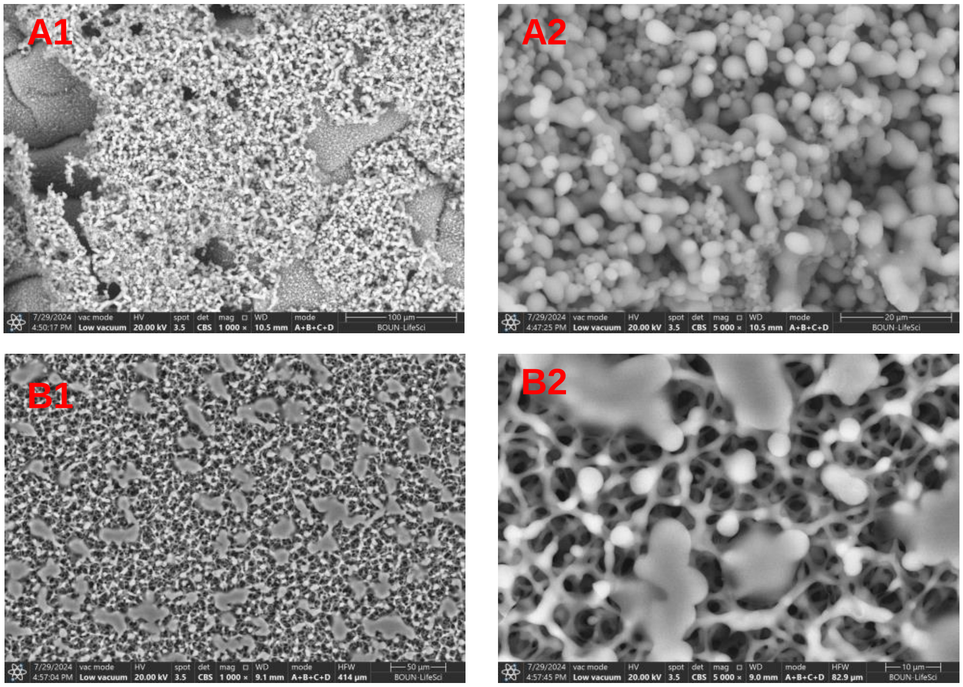

C

Element	Weight %
C	43.2
N	2.8
O	53.1
S	1.0

Element	Weight %
C	45.4
O	54.6
S	0.0

Figure S7. SEM images of hydrogels (A1) PEGDA:HEMA:IBU-CYS 2 (100 µm), (A2) PEGDA:HEMA:IBU-CYS 2 (20 µm), (B1) PEGDA:HEMA (100 µm), (B2) PEGDA:HEMA (20 µm) and (C) EDX analysis
